# Asgard archaea reveal the conserved principles of ESCRT-III membrane remodeling

**DOI:** 10.1126/sciadv.ads5255

**Published:** 2025-02-07

**Authors:** Diorge P. Souza, Javier Espadas, Sami Chaaban, Edmund R. R. Moody, Tomoyuki Hatano, Mohan Balasubramanian, Tom A. Williams, Aurélien Roux, Buzz Baum

**Affiliations:** ^1^ MRC Laboratory of Molecular Biology, Cambridge CB2 0QH, UK.; ^2^ Department of Biochemistry, University of Geneva, CH-1211 Geneva, Switzerland.; ^3^ School of Biological Sciences, University of Bristol, Bristol BS8 1TQ, UK.; ^4^ Centre for Mechanochemical Cell Biology, Division of Biomedical Sciences, Warwick Medical School, University of Warwick, Coventry CV4 7AL, UK.

## Abstract

ESCRT-III proteins assemble into composite polymers that undergo stepwise changes in composition and structure to deform membranes across the tree of life. Here, using a phylogenetic analysis, we demonstrate that the two endosomal sorting complex required for transport III (ESCRT-III) proteins present in eukaryote’s closest Asgard archaeal relatives are evolutionarily related to the B- and A-type eukaryotic paralogs that initiate and execute membrane remodeling, respectively. We show that Asgard ESCRT-IIIB assembles into parallel arrays on planar membranes to initiate membrane deformation, from where it recruits ESCRT-IIIA to generate composite polymers. Last, we show that Asgard ESCRT-IIIA is able to remodel membranes into tubes as a likely prelude to scission. Together, these data reveal a set of conserved principles governing ESCRT-III–dependent membrane remodeling that first emerged in a two-component ESCRT-III system in archaea.

## Teaser

Asgard archaea have two ESCRT-III subunits with the conserved traits that drive stepwise membrane remodeling in eukaryotes.

## INTRODUCTION

All cells and eukaryotic organelles are bound by selectively permeable membranes that must be remodeled and repaired to maintain their organization over time. To aid these processes, cells from across the tree of life use a conserved family of polymer-forming endosomal sorting complex required for transport III (ESCRT-III) proteins that deform, cut, and repair membranes (*1*–*3*). In eukaryotes, ESCRT-III proteins perform a host of important functions at different locations in the cell and in distinct contexts, such as cytokinetic abscission (*4*, *5*), viral budding (*6*), formation of endosomal multivesicular bodies for the degradation of ubiquitinated membrane proteins (*7*), plasma membrane repair (*8*), and nuclear envelope reformation after mitotic exit (*9*, *10*). How the ESCRT-III machinery evolved to work together and achieve these complex membrane remodeling tasks is an important open question.

Eukaryotic genomes encode a multitude of ESCRT-III homologs [12 subunits in humans and more than 25 in some plants (*11*)], which are usually classified into eight subfamilies: Vps46/Did2, Vps2, Vps24, Vps32/Snf7, Vps60, Vps20, CHMP7, and IST1 (CHMP1-7 and IST1 in animals, respectively) (*7*, *12*–*17*). While subunits like CHMP4/Snf7 are known to be active in many cellular contexts (*3*), some have specific functions (*9*, *18*). Nonetheless, many membrane remodeling events require multiple ESCRT-III paralogs acting sequentially and in concert. A well-characterized example of this multistep process is the formation of multivesicular bodies, where CHMP6/Vps20 is recruited to the membrane, followed by CHMP4/Snf7, CHMP2A-CHMP3, and lastly, CHMP1B/IST1 (*7*, *17*, *19*–*22*) In part because of the complexities of the eukaryotic ESCRT-III system, much remains to be understood about the stepwise remodeling of membranes by ensembles of ESCRT-III proteins.

Individual ESCRT-III proteins are small and have a simple set of α helices (α1 to α5) that define the functional core of the protein (*23*, *24*). They can exist in a “closed” conformation, in which the α helices pack up against one another (*25*, *26*) or in an “open” conformation (*27*, *28*). Subunits tend to polymerize in the open conformation, with some exceptions (*27*, *29*, *30*). Polymerization can be aided by membrane binding, in part because ESCRT-III proteins physically associate with negatively charged lipids (*23*, *31*). Since each ESCRT-III homopolymer preferentially binds to membranes of a defined curvature, it is thought that the sequential binding and polymerization of different ESCRT-III subunits progressively reshape flat membranes into tubes (*20*, *32*, *33*). Nevertheless, the specific mechanism by which this occurs remains poorly understood. Here, we investigated the simplified machinery in our closest prokaryotic relatives, the Asgard archaea, to reveal the structural and biophysical features that allow a two-subunit system to perform the tasks of numerous eukaryotic paralogs, underscoring the inherited principles of membrane remodeling by the ESCRT-III machinery.

## RESULTS

### Evolution of Asgard and eukaryotic ESCRT-III

Recent work has shown that homologs of eukaryotic ESCRT-III proteins in archaea perform a range of membrane remodeling functions, including cell division (*34*–*36*), viral budding, and vesicle formation (*37*–*40*). Since archaea have far fewer ESCRT-III homologs than most eukaryotes, we reasoned that their simplified machinery could provide crucial insight into ESCRT-III biology. The most stripped-down version of the ESCRT-III system is found in Asgard archaea, the closest prokaryotic relatives of eukaryotes (*41*–*43*), which have as few as two ESCRT-III homologs (*42*–*44*).

**Fig. 1.**
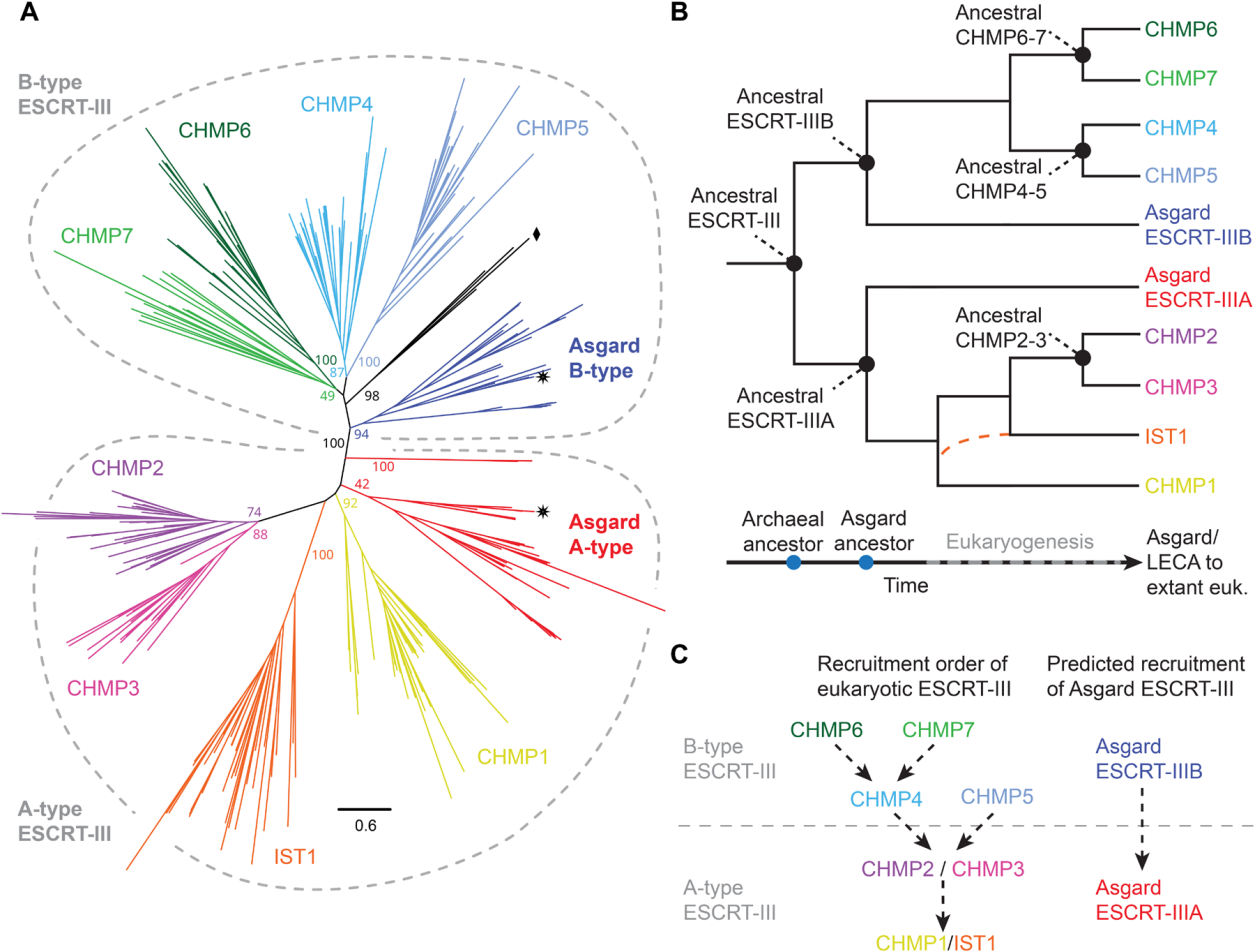
Evolution of the Asgard archaeal/eukaryotic ESCRT-III superfamily. (**A**) Maximum likelihood phylogenetic tree of ESCRT-III proteins in Asgard archaea and eukaryotes. The Asgard A- and B-type proteins are related to eukaryotic CHMP1-3/IST1 and CHMP4-7, respectively. The A- and B-type sequences from Heimdallarchaeota archaeon AB_125 (ESCRT-IIIA and ESCRT-IIIB), investigated experimentally in this study, are labeled with black stars. A small set of eukaryotic sequences lies outside of the main eukaryotic ESCRT-IIIB branch and is labeled with a black diamond. Support values are IQTREE2 ultrafast bootstrap supports (*78*). The scale bar represents the number of amino acid substitutions per site. A complete tree can be found in fig. S1. (**B**) Proposed evolution of the eukaryotic ESCRT-III subfamilies based on the phylogeny shown in (A). The tree topology is consistent with an ancestral gene duplication before the Asgard ancestor, followed by several rounds of gene duplication during eukaryogenesis, giving rise to all CHMP1-7/IST1 paralogs in the eukaryotic lineage before the last eukaryotic common ancestor (LECA). A proposal of when each ancestral ESCRT-III form appeared or existed is presented at the bottom. The position of IST1 in the eukaryotic A-type branch is not certain and could have followed pathways indicated by black or orange lines. euk., eukaryotes. (**C**) The recruitment order of eukaryotic ESCRT-III proteins to membranes is related to the evolutionary history of its subfamilies. Taking into account the phylogeny (A and B) and the described recruitment order of their eukaryotic homologs [(C), left], this analysis predicts that Asgard ESCRT-IIIB binds flat membranes and recruits ESCRT-IIIA [(C), right].

We began by investigating the evolutionary relationship between Asgard and eukaryotic homologs, including representatives of the complete set of eukaryotic ESCRT-III protein subfamilies. A phylogenetic analysis revealed the existence of two main, maximally supported (100% bootstrap) clades of ESCRT-III proteins, each containing one Asgard ESCRT-III paralog (ESCRT-IIIA and ESCRT-IIIB) and several eukaryotic subfamilies (Fig. 1A and fig. S1) (*42*, *43*). The ESCRT-IIIA family (hereafter A-type) contains CHMP1/Vps46/Did2, CHMP2/Vps2, CHMP3/Vps24, and IST1, while the ESCRT-IIIB family (hereafter B-type) contains CHMP4/Vps32/Snf7, CHMP5/Vps60, CHMP6/Vps20, and CHMP7/CHM7 (*1*, *42*, *45*). The topology of the tree suggests a simple model of ESCRT-III evolution in which an ancestral gene duplicated in the common ancestor of all Asgard archaea, giving rise to two specialized ESCRT-III proteins (Fig. 1B). Over the course of several rounds of gene duplication and diversification during eukaryogenesis, these paralogs gave rise to the complete eukaryotic set (Fig. 1B).

Notably, an inspection of the eukaryotic subfamilies within the phylogenetic tree suggests that the ESCRT-III proteins within each of the two sister clades share functional and structural roles with one another. B-type proteins are typically recruited earlier in the membrane-remodeling pathway (CHMP4/5/6/7), while A-type proteins are recruited later (CHMP1/2/3/IST1) (Fig. 1C). Therefore, this order may stem from an early adaptation and might still be found in the two ESCRT-III homologs of Asgard archaea, with one acting “before” (ESCRT-IIIB) and one acting “after” (ESCRT-IIIA) (Fig. 1C). To test whether the recruitment order predates eukaryogenesis and how two Asgard paralogs can perform the work of their several eukaryotic counterparts, we investigated the structural and biophysical properties of ESCRT-III proteins encoded by one of our closest Asgard relatives, Heimdallarchaeota archaeon AB_125 (hereafter Asgard) (*41*, *43*).

### Structure of the Asgard ESCRT-IIIB protofilament

**Fig. 2.**
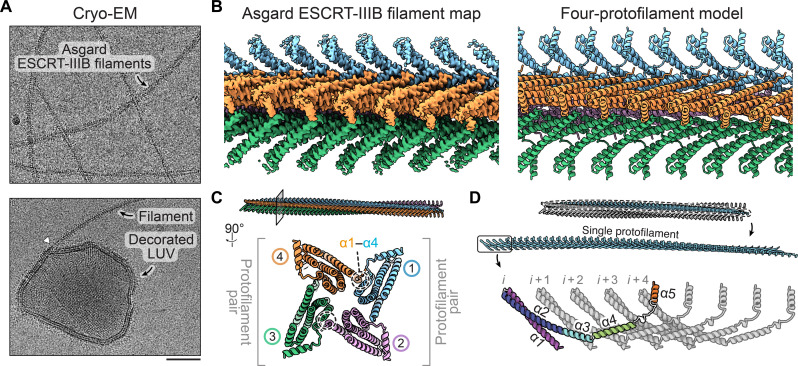
Asgard ESCRT-IIIB filament structure. (**A**) Cryo–electron micrographs of Asgard ESCRT-IIIB filaments in the presence of LUVs. The bottom micrograph shows an example of a decorated LUV. An instance of a filament connecting to a LUV is highlighted by the white triangle (scale bar, 50 nm). (**B**) Density map (left) and model (right) of the Asgard ESCRT-IIIB filament. The polymers are composed of four protofilaments (blue, pink, green, and orange). (**C**) Top view of a cross section through the filament, highlighting the two protofilament pairs and the interprotofilament interactions through α1 and α4. (**D**) Domain structure of the Asgard ESCRT-IIIB protofilament. Five subunits are shown (*i* to *i* + 4). Secondary structure elements (helices α1 to α5) of subunit *i* are highlighted.

While much is known about the structure of A-type ESCRT-III subunits and protofilaments, the only known structures for B-type proteins are truncated versions of Snf7/CHMP4 solved by x-ray crystallography (*28*, *46*) and Snf7 protofilament spirals imaged by cryo–electron microscopy (cryo-EM), which resolved the α1 to α3 secondary structures (*47*). Therefore, to understand how B-type protofilaments are constructed, we began by purifying recombinant full-length ESCRT-IIIB from Asgard (fig. S2A), which was soluble and mostly monomeric (fig. S2B). However, after mixing the protein with large unilamellar vesicles (LUVs) containing 40 mol % of the negatively charged lipid 1,2-dioleoyl-*sn*-glycero-3-phospho-l-serine (DOPS), long filaments were observed by cryo-EM (Fig. 2A). These filaments have a diameter of ~12 nm and are reminiscent of those formed by eukaryotic B-type ESCRT-III proteins (*48*–*50*). Filaments were also seen decorating and occasionally interacting with LUVs of various shapes, further suggesting that polymer nucleation is aided by the presence of membranes under these conditions (Fig. 2A).

The regularity of the filaments allowed us to solve their structure using single-particle analysis and helical reconstruction to a resolution of 2.9 Å (Fig. 2B; fig. S3, A and B; and table S1). Filaments were found to consist of four closely associated, parallel protofilaments arranged in two pairs with a shallow twist (~1.5°) (Fig. 2C and fig. S3C). Each protofilament is built from monomers that interact through α helices α1 to α5 (Fig. 2D and fig. S3C). Protofilaments are bound to their neighbors via electrostatic interactions linking helices α1 (N terminus) and α4 (fig. S3D). The region of α4 mediating this interaction differs between adjacent pairs, resulting in a 1.5-nm offset (fig. S3, C and D).

The monomers in these protofilaments adopt an open configuration (fig. S3C). Each monomer (*i*) makes extensive contacts with its neighbors at positions *i* + 1, *i* + 2, *i* + 3, and *i* + 4 (fig. S3E), as described for eukaryotic A-type subunits (*27*, *51*). This differs from the bacterial ESCRT-III homologs PspA and Vipp1, where interactions between subunits in a protofilament reach from *i* to *i* + 3 while maintaining the highly conserved interaction between α5 of subunit *i* and the hairpin α1/α2 (*1*, *52*, *53*). Therefore, the underlying structural characteristics of ESCRT-III protofilaments are conserved from archaea to eukaryotes.

### Membrane binding dynamics of Asgard ESCRT-IIIB

**Fig. 3.**
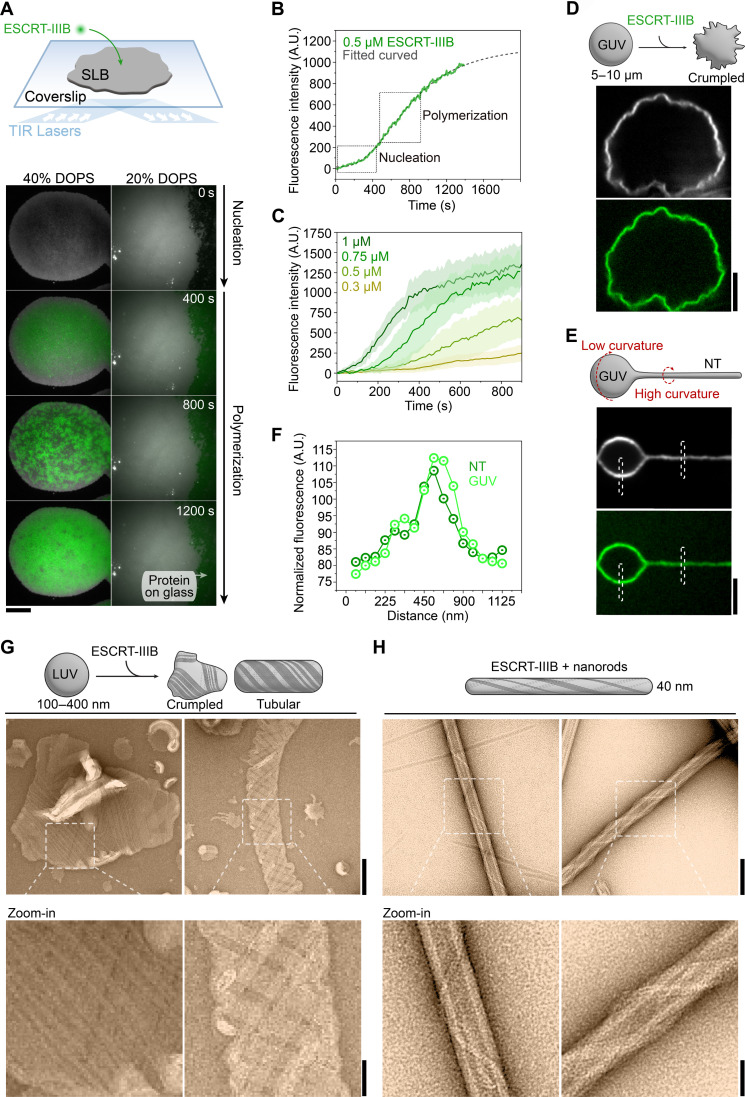
Interaction of Asgard ESCRT-IIIB with membranes. (**A**) Schematic of the setup in which time-lapse TIRF microscopy is used to follow ESCRT-IIIB (green) recruitment to flat SLBs (gray). Left and right columns show membranes containing 40 and 20 mol % DOPS, respectively. ESCRT-IIIB binds and self-assembles on 40 mol % DOPS–containing SLBs and fails to interact with 20 mol % DOPS–containing SLBs. Scale bar, 2.5 μm. (**B**) Fluorescence time profile of 0.5 μM ESCRT-IIIB recruitment to an SLB. The green line shows ESCRT-IIIB mean fluorescence intensity (*n* = 9 biological replicates) measured on 40 mol % DOPS membranes [as shown in (A)]. Left and right dashed boxes indicate ESCRT-IIIB nucleation and polymerization, respectively. The dashed gray line shows the fitting curve using the Hill function. A.U., arbitrary units. (**C**) Fluorescence time profiles of ESCRT-IIIB (0.3, 0.5, 0.75, and 1 μM) measured on 40 mol % DOPS SLBs (means and SD are shown; *n* = 9 biological replicates). (**D**) Schematic of ESCRT-IIIB added to a GUV and fluorescence image of ESCRT-IIIB–induced membrane crumpling. Scale bar, 3 μm. (**E**) Schematic of a GUV with a pulled NT. Fluorescence image of ESCRT-IIIB binding to a giant unilamellar vesicle (GUV) with a pulled membrane nanotube (NT). Dashed boxes show where the fluorescence plot profile was obtained to measure preferential protein recruitment dependent on membrane curvature. In (D) and (E), the protein and membrane fluorescence signals are shown in green and gray, respectively. Scale bar, 2 μm. (**F**) Fluorescence profiles of ESCRT-IIIB on a GUV and lipid NT normalized to the membrane fluorescence of areas shown in (E). (**G**) Negative-stain EM of ESCRT-IIIB filaments wrapping around flat and tubular LUVs and (**H**) nanorods. Scale bars are 100 and 40 nm in the zoom-out and zoom-in images, respectively.

The requirement of LUVs for filament formation suggests that membranes trigger ESCRT-IIIB self-assembly. To directly observe this process, we fluorescently labeled ESCRT-IIIB and visualized its recruitment to supported lipid bilayers (SLBs) using total internal reflection fluorescence (TIRF) microscopy (Fig. 3A). While yeast Snf7 (B-type) can nucleate and polymerize on membranes composed of 20 mol % of negatively charged DOPS (*50*), this was insufficient to recruit Asgard ESCRT-IIIB (Fig. 3A). However, robust nucleation and growth of 0.5 μM ESCRT-IIIB were observed when SLBs contained ≥40 mol % DOPS (Fig. 3A). On these DOPS-rich SLBs, Asgard ESCRT-IIIB initially formed a homogeneous layer within which small punctae appeared and grew until they completely covered the membrane. These data indicate that electrostatic interactions between ESCRT-IIIB and negatively charged head groups of lipids in membranes are important for nucleation, as they are for Snf7 (*50*).

An analysis of the fluorescence increase over time revealed a sigmoidal curve (Fig. 3B), suggesting a two-step kinetic process (*54*, *55*), with an initial lag phase (rate-limiting nucleation) followed by a rapid growth phase (monomer addition), until a slowdown occurs as membrane coverage reaches saturation (Fig. 3B). ESCRT-IIIB therefore polymerizes on membranes in a manner similar to other cytoskeletal polymers like actin (*54*). Consistent with this interpretation, the process was concentration dependent (Fig. 3C). Below 200 nM, only a small uniform fluorescence signal was observed on the membrane, indicative of monomer binding without nucleation (fig. S4A). The growth rate of the polymers was proportional to the monomer concentration (Fig. 3, B and C; and fig. S4, B and C), indicating an absence of cooperativity in the polymerization process. The final steady-state fluorescence intensities were similar regardless of the monomer concentration (fig. S4, D and E), suggesting that it is surface accessibility rather than bulk depletion that limits further polymer assembly.

To understand how the self-assembly of Asgard ESCRT-IIIB affects membrane geometry, giant unilamellar vesicles (GUVs) were mixed with the protein at levels greater than the critical concentration required for self-assembly, resulting in complete decoration by the protein. Under these conditions, >30% of GUVs exhibited a “crumpled” appearance (Fig. 3D), which was not seen with lower membrane coverage of ESCRT-IIIB (fig. S4F). This “crumpling” resembles the effect of Snf7 on GUVs (*50*) and is likely due to the formation of a dense, flat, and continuous protein coat on the membrane (Fig. 2A) that compresses the GUV without imposing a specific curvature on it.

B-type proteins like Snf7 are preferentially recruited to flat membranes (*50*, *56*) and tend to be excluded from the high curvature of membrane nanotubes (NTs) (*20*, *50*, *56*). To test whether Asgard ESCRT-IIIB acts in this way, we pulled membrane NTs from spherical GUVs and compared ESCRT-IIIB fluorescence intensity on different parts of the membrane (Fig. 3E). Notably, while ESCRT-IIIB bound low-curvature GUVs, acting in a similar manner to Snf7, it also bound to highly curved NTs (radius of curvature of >1000 nm in the spherical GUV versus ~50 nm in the pulled NTs). Moreover, when normalized to the membrane intensity, levels of ESCRT-IIIB fluorescence appeared comparable in both regions of the membrane (Fig. 3F). These data suggest that Asgard ESCRT-IIIB polymers can adapt to a wide range of membrane curvatures.

To understand how ESCRT-IIIB can bind membranes of higher curvature, we performed negative-stain EM to visualize its association with LUVs (100 to 400 nm in diameter) and thin cylindrical galactocerebroside nanorods (~40 nm in diameter) (fig. S4, G and H) (*57*, *58*). On LUVs, ESCRT-IIIB polymers formed large lattices with angular edges (Fig. 3G), consistent with the “crumpled” structures seen on larger GUVs (Fig. 3D). In addition, we observed instances in which the LUVs formed tubes with tilted polymer arrays, resembling a mesh in the projected two-dimensional (2D) images (Fig. 3G). Conversely, when ESCRT-IIIB was added to nanorods, which have higher curvature, filaments were able to wrap around the membrane by adopting a shallow angle (Fig. 3H), resembling the curved filaments formed by eukaryotic B-type mixed with A-type proteins (Snf7-Vps2-Vps24) (*59*). This is unlike eukaryotic B-type proteins Snf7 and CHMP4B, which either do not bind tubes smaller than 75 nm (*20*) or lie parallel to the tube axis (*56*). These data confirm that, in contrast to its eukaryotic counterparts, the polymers formed by Asgard ESCRT-IIIB have the structural flexibility that allows them to accommodate a wide range of membrane curvatures (fig. S4I) (*49*, *50*).

### Structure of Asgard ESCRT-IIIB membrane arrays

**Fig. 4.**
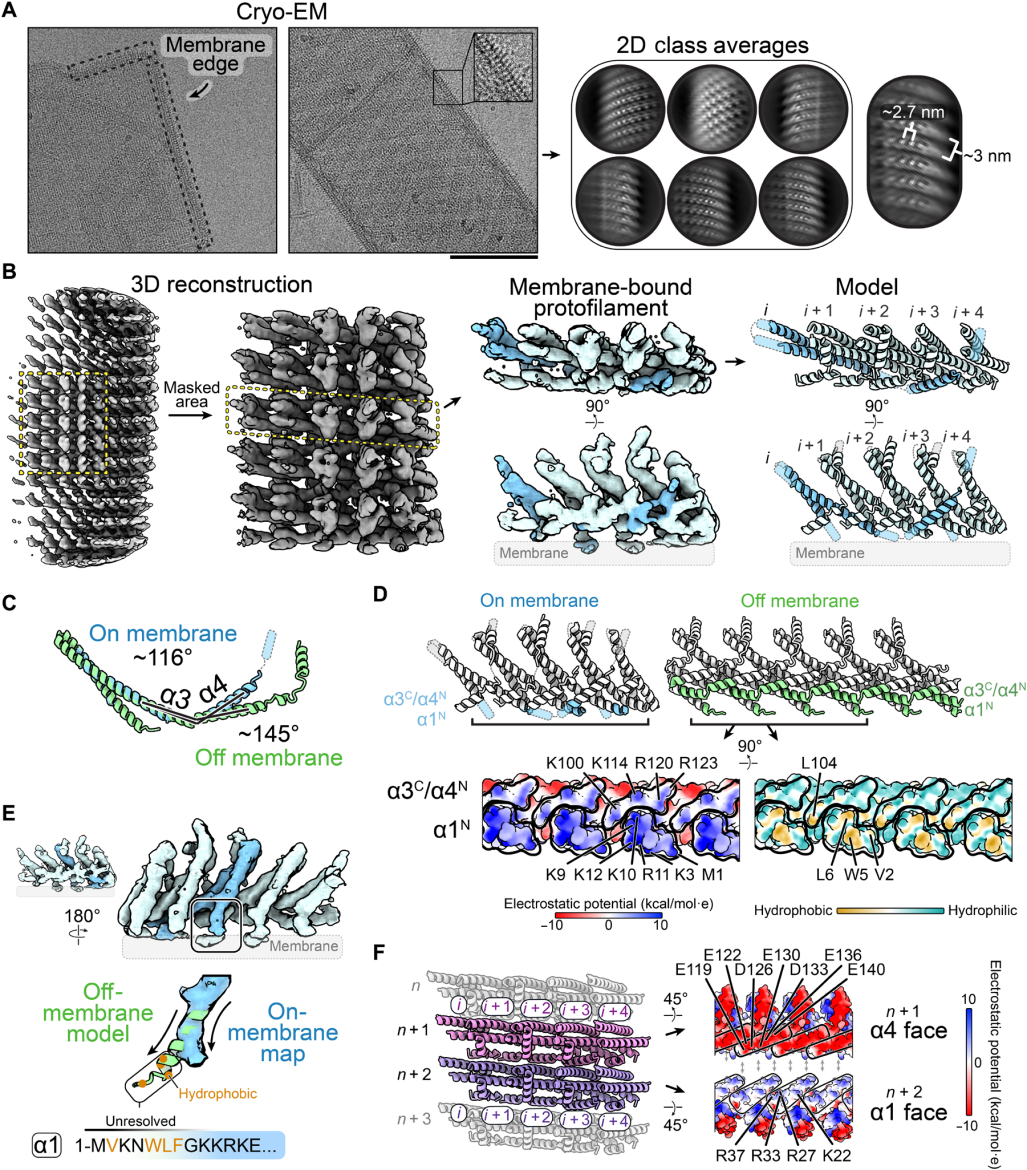
Structure of Asgard ESCRT-IIIB bound to membranes. (**A**) Cryo–electron micrograph of Asgard ESCRT-IIIB decorating LUVs, with repeating spikes at the membrane edge (left; scale bar, 100 nm). Picked particles along the membrane edge are used for 2D classification (right). (**B**) Density map of Asgard ESCRT-IIIB bound to membranes, showing the area masked for refinement (left). A single protofilament is highlighted along with the fit model (right). (**C**) Overlay of the on-membrane and off-membrane models after aligning at α1 to α3, highlighting the angle between α3 and α4. (**D**) Bottom view of the high-resolution Asgard ESCRT-IIIB off-membrane protofilament structure showing the charged/hydrophobic residues that would be exposed in the on-membrane model. (**E**) Rotated view of the protofilament on membranes showing a deviating helix α1 and unresolved N terminus, where a hydrophobic patch may extend into the membrane. (**F**) Model of the polymeric arrangement of several Asgard ESCRT-IIIB protofilaments on LUVs (left). Lateral interactions between protofilaments are mediated by complementarily charged surfaces in helices α1 (blue; positively charged surface) and α4 (red, negatively charged) (right). Some of the key residues at the interface have been highlighted.

To begin understanding the structural basis for these properties, we attempted to solve the structure of Asgard ESCRT-IIIB filament arrays bound to membranes (fig. S5A). Because of the presence of the membrane signal, we were unable to accurately align the bound protein. Therefore, we devised a strategy to reduce the membrane signal in micrographs (fig. S5A), inspired by studies on microtubule decoration by dynein motors (*60*, *61*). This allowed us to more clearly define the protofilament arrays (Fig. 4A). Despite their structural variability, we could solve the structure of the arrays to a 6.5-Å resolution, allowing us to confidently assign α helices (Fig. 4B; fig. S5, B and C; and table S1).

A comparison with our off-membrane structure revealed a slightly smaller intersubunit distance in the membrane-bound protofilament (~3 nm versus 2.7 nm, respectively). This was accompanied by a change in the angle between helices α3 and α4 (off-membrane: 145°; on-membrane: 116°) (Fig. 4C). This indicates that the loop between α3 and α4 acts as a hinge aiding the conformational flexibility of the protofilament, which is consistent with previous reports from eukaryotic and bacterial ESCRT-III polymers (*1*, *24*, *29*, *62*). The conformational differences between the membrane-bound and unbound polymers may be influenced by the distinct interactions therein, underscoring the adaptability of the ESCRT-IIIB protofilament.

The structure of membrane-bound Asgard ESCRT-IIIB also revealed that the protein binds membranes via the N terminus of α1 and the loop connecting helices α3 and α4 (Fig. 4D). This interface includes an exposed region of continuous positive charge as well as hydrophobic residues (Fig. 4D), consistent with the binding of filaments to negatively charged membranes (Fig. 3A). This region of α1 mediates the interaction between protofilaments in our off-membrane structure, although interacting with the negatively charged surface of α4 (fig. S3D). By superimposing our high-resolution structure onto the membrane-bound map, we noticed that hydrophobic residues of helix α1 were positioned to insert into the lipid core of the membrane (Fig. 4E). While this interface differs from the reported interaction site of CHMP1B (A-type) with membranes (*29*, *63*), it is similar to the membrane binding interface of Snf7 (B-type) (*47*) and CHMP2A-CHMP3 (A-type) (*51*).

An analysis of adjacent membrane-bound protofilaments revealed that interactions between them are mediated by their planar faces through the positive charges of helix α1 and negative charges of α4 (Fig. 4F). Helical filaments formed by CHMP2A-CHMP3 use similar charge complementarity to mediate interactions between adjacent rungs (*51*). Equivalent lateral interactions have also been proposed to connect B-type/A-type composite polymers (Snf7 and Vps24) (*64*). Therefore, complementary charges located on extended surfaces are a general feature of B- and A-type homo- and heteropolymers that may enable protofilaments to flexibly associate with one another in ways that allow for sliding (*64*). Together, these data suggest that ESCRT-IIIB subunits and the protofilaments they form have intrinsic flexibility that allows them to bind membranes of different curvatures.

### Recruitment of ESCRT-IIIA to membranes by ESCRT-IIIB

**Fig. 5.**
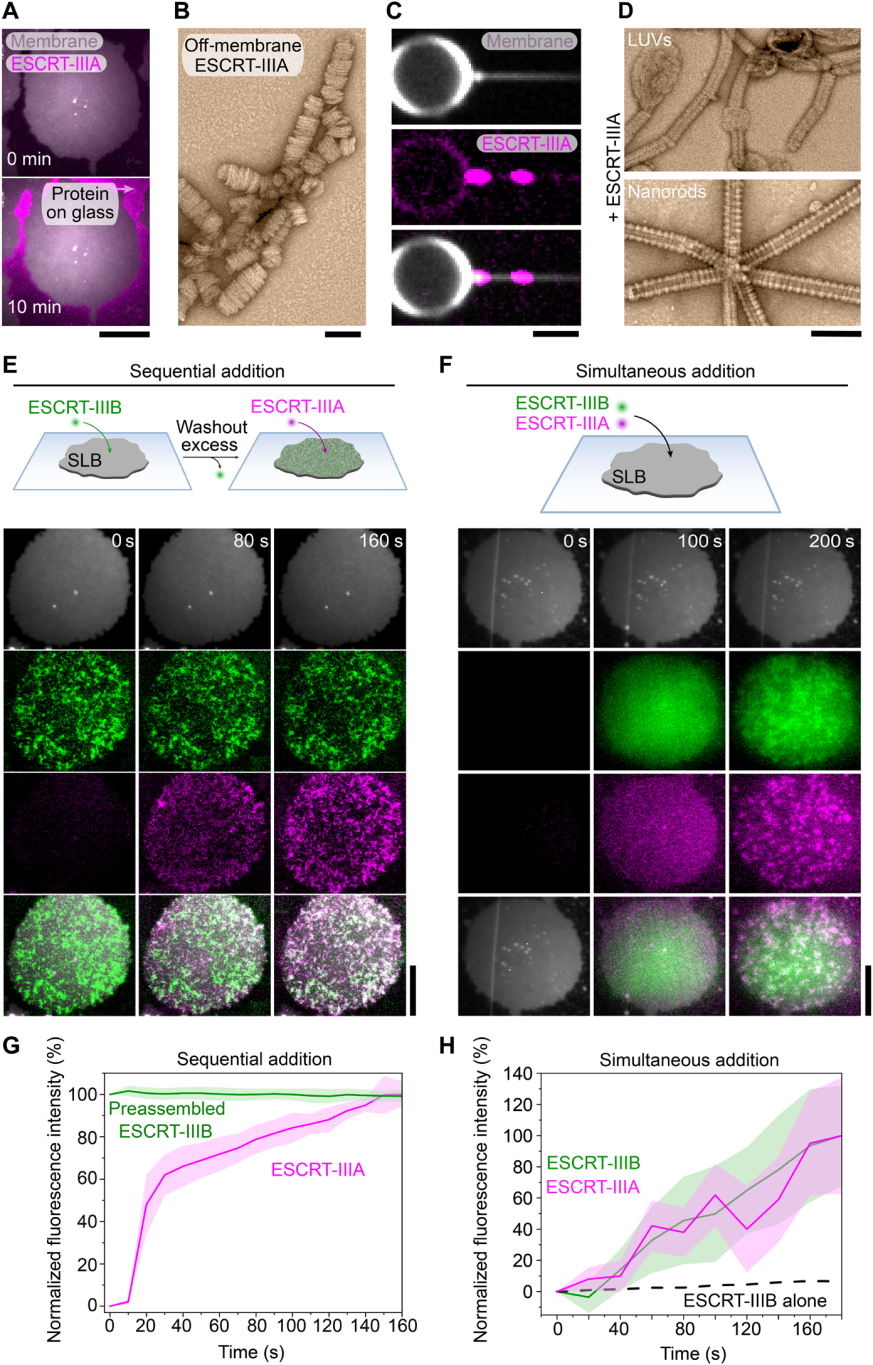
Characterization of Asgard ESCRT-IIIA and its recruitment to flat membranes. (**A**) Representative TIRF microscopy image of 1 μM Asgard ESCRT-IIIA added to 40 mol % DOPS SLB membranes after 10-min incubation. Scale bar, 2.5 μm. (**B**) Negative-stain electron micrographs of Asgard ESCRT-IIIA helical assemblies without membranes. Scale bar, 100 nm. (**C**) Fluorescence image of ESCRT-IIIA forming clusters on the highly curved part of the membrane NT (pink; middle) that was pulled from a GUV (gray; top) after 15-min incubation and washing. Merge is shown at the bottom. Scale bar, 1 μm. (**D**) Negative-stain electron micrographs of Asgard ESCRT-IIIA on LUV and nanorod membranes. ESCRT-IIIA forms a helical assembly on nanorods (bottom) and induces tubulation on LUVs (top). Scale bar, 110 nm. (**E**) Representation of the sequential addition experiment. Fluorescence time-lapse images using TIRF microscopy to show ESCRT-IIIA recruitment to the SLB membranes with pre-assembled ESCRT-IIIB patches. Scale bar, 2.5 μm. ESCRT-IIIB (1 μM) was added to form the patches and washed out before complete membrane coverage, and then 1 μM ESCRT-IIIA was added. (**F**) Representation of the simultaneous addition experiment. Fluorescence time-lapse images using TIRF microscopy show how ESCRT-IIIB and ESCRT-IIIA were recruited to SLBs (1 μM of each protein). Scale bar, 2.5 μm. (**G**) Fluorescence-based quantification of ESCRT-IIIB and ESCRT-IIIA signals over time of the experiment shown in (E) (lines represent the mean fluorescence intensity, and shades are the SD; *n* = 9 biological replicates). (**H**) Dynamics of ESCRT-IIIB recruitment when added to the microscopy chamber alone (black dashed line: mean fluorescence; *n* = 9 biological replicates) or together with ESCRT-IIIA (green line and shade, mean fluorescence and SD, respectively; *n* = 7 biological replicates). ESCRT-IIIA recruitment kinetics are shown in magenta (line and shade, mean fluorescence and SD, respectively; *n* = 9 biological replicates).

The observation that Asgard ESCRT-IIIB binds flat membranes is consistent with its role in initiating membrane remodeling, as suggested by our phylogenetic analysis (Fig. 1C). In line with this, when we performed similar experiments with fluorescently labeled Asgard ESCRT-IIIA, it could not bind SLBs, even at high concentrations (Fig. 5A and fig. S2A). We suspected that the inability of Asgard ESCRT-IIIA to bind flat membranes was due to an intrinsic preference of ESCRT-IIIA for curved membranes. Supporting this notion, ESCRT-IIIA formed thin, irregular helical filaments in the absence of membranes (Fig. 5B), like its eukaryotic A-type homologs (*27*, *29*, *51*, *56*, *59*). Moreover, while fluorescently labeled ESCRT-IIIA bound negligibly to the body of GUVs, it could self-assemble into punctae on the highly curved pulled NTs (~20× smaller diameter) (Fig. 5C). When ESCRT-IIIA was added to LUVs or nanorods, it formed filaments that wrapped around the membrane, with diameters of 31.06 ± 2.69 and 43.18 ± 2.21 nm, respectively (Fig. 5D and fig. S6A), similar to A-type CHMP1B (~28 nm) (*29*). Thus, while Asgard ESCRT-IIIB polymers can bind membranes with a wide range of curvatures, ESCRT-IIIA preferentially binds membranes with high curvature.

To directly test whether ESCRT-IIIB can recruit ESCRT-IIIA to flat membranes, we first coated flat SLBs with ESCRT-IIIB and washed away the excess. Consistent with this hypothesis, when we then added ESCRT-IIIA, it specifically bound to ESCRT-IIIB patches (Fig. 5E). Moreover, when the two proteins were added simultaneously to SLBs, similar patches containing both proteins appeared (Fig. 5F), with kinetics resembling those of ESCRT-IIIA on preassembled ESCRT-IIIB (~160 s to reach plateau) (Fig. 5G). This was much faster than the rates observed for ESCRT-IIIB alone (>1000 s to reach plateau) (Fig. 5H and fig. S6B), indicating that the two proteins act synergistically to bind and copolymerize on membranes.

**Fig. 6.**
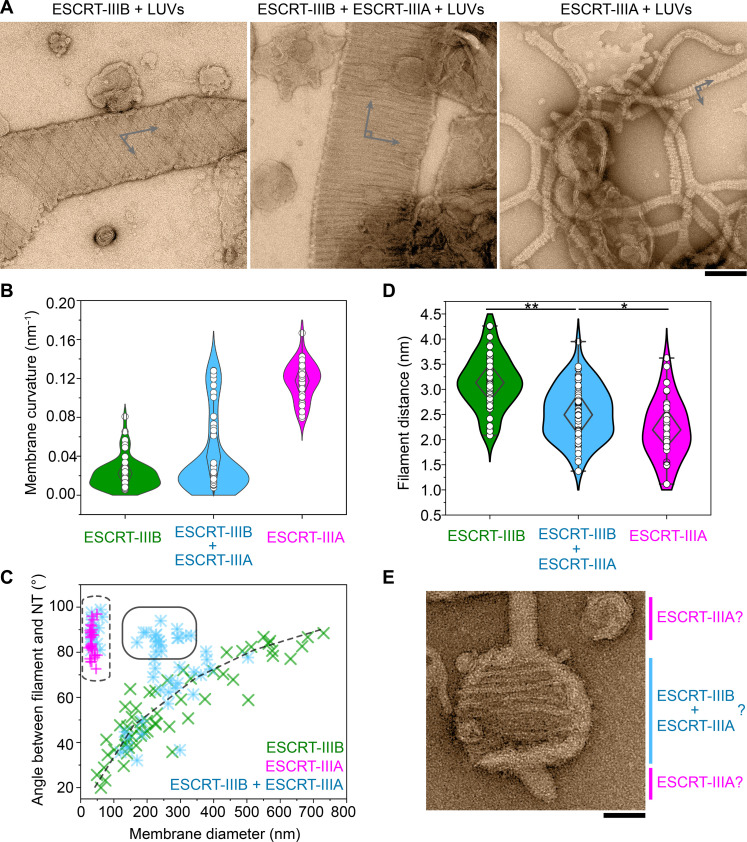
Composite polymers generated by the coaddition of ESCRT-IIIB and ESCRT-IIIA to membranes. (**A**) Representative negative-stain electron micrographs of ESCRT-IIIB alone (left), the ESCRT-IIIB-ESCRT-IIIA mixture (middle), and ESCRT-IIIA alone (right) upon incubation with membranes. Scale bar, 150 nm. Drawn lines over the micrographs indicate the angle taken to calculate the filament orientation shown in (C). (**B**) Curvature estimates of the tubulated membranes decorated with ESCRT-IIIB (*n* = 57), ESCRT-IIIA (*n* = 30), and ESCRT-IIIB and ESCRT-IIIA mixtures (*n* = 57) analyzed from electron micrographs. (**C**) Measurement of filament orientation defined by the angle between the membrane and the filament axis [shown in (A)]. Green and magenta crosses indicate experiments including only ESCRT-IIIB (*n* = 57) or ESCRT-IIIA (*n* = 30) in the presence of membranes, respectively. Light blue asterisks indicate orientations measured for the ESCRT-IIIB and ESCRT-IIIA mixed sample (*n* = 57) with membranes. The dashed box and line represent the ESCRT-IIIA and ESCRT-IIIB populations, respectively. The solid box outlines the population that is distinct in the ESCRT-IIIB/A mixture. (**D**) Interfilament distance measured from electron micrographs of ESCRT-IIIB (*n* = 58), ESCRT-IIIA (*n* = 43), and the mixture of ESCRT-IIIB and ESCRT-IIIA (*n* = 63) decorating membranes (statistical significance, unpaired two-tailed *t* test; *P* values are 0.0001 and 0.02428 from left to right). (**E**) Representative micrograph obtained by negative-stain EM showing the membrane curvature in the presence of ESCRT-IIIA and ESCRT-IIIB, transitioning between less curved (likely decorated with both proteins) and highly curved (likely decorated with only ESCRT-IIIA filaments) regions. Scale bar, 50 nm. In all cases, *n* shows biological replicates.

Previous in vitro and in silico studies have suggested that such sequential binding of ESCRT-III proteins with different curvature preferences can alter both membrane curvature (*20*) and filament structure (*32*). Furthermore, molecular dynamics simulations predict that ESCRT-III heteropolymers transform flat membranes into tubes as they pass through intermediate states (*32*, *65*, *66*). We therefore tested whether such a graded shift in structure occurs in a two-subunit system. When mixed with LUVs, ESCRT-IIIB promoted lower membrane curvature than ESCRT-IIIA, as expected (Fig. 6, A and B). Mixing both subunits together resulted in a distribution of membrane curvatures that spanned the range observed with ESCRT-IIIA or ESCRT-IIIB alone (Fig. 6B). When we analyzed the filament orientation as a function of membrane diameter, a distinct population appeared in the ESCRT-IIIB/A mixture, which shares both the lower membrane curvature seen with ESCRT-IIIB and the perpendicular filament orientation seen with ESCRT-IIIA (Fig. 6, A and C). This population additionally exhibited a reduction in the average distance separating adjacent protofilaments in the arrays (2.53 ± 0.53 nm, down from 3.13 ± 0.53 nm with ESCRT-IIIB alone) (Fig. 6D). Although the data do not allow us to determine the local filament composition, this population may represent ESCRT-IIIA/B copolymers. Moreover, in the ESCRT-IIIA/B mixtures, we could find rare cases of different membrane curvatures on the same LUV (Fig. 6E), potentially capturing distinct steps in the remodeling pathway. These data collectively illustrate how stepwise changes in the composition of ESCRT-III polymers translate into alterations in filament geometry and an increase in membrane curvature before scission can occur.

## DISCUSSION

Since the discovery of ESCRT-III proteins in eukaryotes (*7*, *12*), numerous studies have examined their structure and biochemistry to help explain how they remodel membranes. There is interesting variability in these data, including polymers that contain subunits in the open or closed conformation (*27*, *29*, *30*), have different membrane binding interfaces (*27*, *29*, *63*), and engage with positively and negatively curved membranes (*3*). Despite this variability, these studies have revealed that (i) most ESCRT-III polymers have subunits in the open conformation, which engage in intimate interactions over a distance of three to four subunits along the length of the protofilament; (ii) they can simultaneously associate with negatively charged lipids and with one another to generate 2D and 3D membrane coats; (iii) differences in the structure and flexibility of individual ESCRT-III monomers dictate the curvatures their protofilaments can adopt; (iv) different ESCRT-III proteins form composite polymers with intermediate curvatures that help to remodel flat membranes into tubes (*33*).

Here, through our analysis of a simple two-subunit ESCRT-III system identified in one of our closest prokaryotic relatives, Heimdallarchaeaota, we demonstrate that eukaryotes inherited all these structural features from their archaeal ancestors. Our phylogenetic analysis also shows that the entire set of eukaryotic ESCRT-III proteins can be organized into two families, which we term the B-type (before) and A-type (after) proteins. This organization can be found in the two subunits encoded by the genomes of Asgard archaea, ESCRT-IIIB and ESCRT-IIIA, which act in sequence to transform a flat membrane into a tube, an activity that, in eukaryotes, requires a much larger set of ESCRT-III proteins.

### ESCRT-IIIB flexibility and membrane interaction

Our structural analysis of Asgard ESCRT-IIIB, both on and off membranes, constitutes the first detailed structure of a B-type polymer built from full-length subunits (fig. S7) (*28*, *46*, *47*). We show how protofilaments are able to adapt to different membrane curvatures, likely through the intrinsic flexibility of ESCRT-IIIB at the hinge between helices α3 and α4. These properties help recapitulate the membrane binding and remodeling capabilities of B-type eukaryotic ESCRT-III proteins and some eukaryotic B-type/A-type composites. The structure also reveals how protofilaments interact with membranes using an interface similar to other eukaryotic B- and A-type proteins (*47*, *51*). Hydrophobic residues at the N terminus of ESCRT-III proteins may dip into the lipid bilayer, suggesting that this region also contributes to membrane remodeling. The positively charged interface of helix α1 that mediates lateral interactions between adjacent protofilaments could act as a second membrane binding region, similar to the one used by CHMP1B polymers (*27*, *29*, *63*). Switching between these two binding modes may help remodel membranes (*32*).

### Interplay between Asgard ESCRT-III and membranes

Although the precise nature of Asgard cell membranes has yet to be determined, our data reveal a requirement for a critical concentration of ESCRT-IIIB protein to initiate polymer formation on reconstituted membranes. This likely reflects the need to form a stable five-subunit nucleus involving *i* to *i* + 4 interactions, which then extends by fast polymerization, as is the case for other cytoskeletal elements like actin (*67*). In cells, it is likely that this nucleation barrier can be overcome by the local accumulation of other ESCRT components at sites rich in ubiquitinated proteins, since Asgard have components equivalent to the eukaryotic multivesicular body machinery, which includes ubiquitin, ubiquitin modification enzymes, and ESCRT-I/II homologs (*42*–*44*).

Our reconstitution experiments show that, although Asgard ESCRT-IIIA can bind by itself to highly curved membranes, it is only recruited to flat membranes in the presence of ESCRT-IIIB. This is similar to what is seen when combining eukaryotic B- and A-type ESCRT-III proteins (*68*) and shows that interactions between the two protein classes and the membrane impose order on the sequence of membrane remodeling events. In contrast to eukaryotes, however, we did not observe helical tubes (*56*, *59*), suggesting that a stepwise change in filament orientation in a simplified two-subunit system may be sufficient to remodel the membrane. In addition, our data reveal unexpected positive feedback in the system, since, when added simultaneously, the ESCRT-IIIA recruited by ESCRT-IIIB can in turn increase the rate of ESCRT-IIIB recruitment, leading to the rapid formation of an ESCRT-III coat. Notably, membranes treated with both ESCRT-III proteins were found to be of intermediate curvature, consistent with the transformation of a flat membrane into one with a shallow buckle (*32*). Once the membrane has been deformed in this way, the potential disassembly of ESCRT-IIIB by Asgard Vps4 (*44*) may allow ESCRT-IIIA to reach its preferred radius of curvature, constricting the membrane into a tube before scission can occur.

### Evolution of the ESCRT-III machinery

Our work shows that Asgard ESCRT-IIIB and ESCRT-IIIA provide an excellent simple model of multicomponent ESCRT-III–dependent membrane remodeling in which B-type proteins, like CHMP4B and CHMP6, recruit A-type proteins, like CHMP2A-CHMP3 and CHMP1B-IST1, to transform a flat membrane into a tube. This shows that a two-protein, multistep membrane remodeling process by ESCRT-III was already established in Asgard archaea before mitochondrial endosymbiosis and the origin of eukaryotes. It should be noted, however, that the cell biological function of ESCRT proteins in Asgard archaea remains to be determined. This is because it is only recently that the first members of the Asgard archaea (Lokiarchaeota) were identified and grown in culture (*69*, *70*). Imaging revealed that the Lokiarchaeota present in these cultures possess a single bounding membrane, long membrane protrusions which may physically contact their symbiotic partners, and generate extracellular vesicles. It is not clear which cytoskeletal proteins are involved in the formation of the protrusions in Asgard cells, nor whether ESCRT-III proteins are likely to play a role in their formation or loss. It is noticeable, however, that ESCRT-III genes in both Heimdallarchaeota and Lokiarchaeota genomes are found in gene clusters coding for ubiquitin and ubiquitin-related machinery, together with ESCRT-I and ESCRT-II (*44*). Since this machinery works together to generate intralumenal multivesicular bodies in eukaryotes, ESCRT-III in Asgard archaea may act in a similar way to rid cells of excess or damaged ubiquitinated membrane proteins through the generation of extracellular vesicles.

In summary, this interdisciplinary analysis of Asgard ESCRT-III proteins clarifies the picture of ESCRT-III–dependent membrane remodeling across the tree of life. Together with other data, this leads us to propose a model for ESCRT-III evolution: All ESCRT-III proteins emerged from a single protein that performed a membrane repair/remodeling function in the last common ancestor of bacteria and archaea (*1*). This protein was modified over time—leading to the evolution of PspA/Vipp1 in bacteria and a multisubunit ESCRT-III in archaea. In the common ancestor of Asgard archaea and eukaryotes, the system became further elaborated through the evolution of distinct B- and A-type ESCRT-III polymers that act in sequence. During eukaryogenesis, further complexification may have emerged as a simple consequence of the expansion in the size of the eukaryotic genome (*71*). At the same time, the increase in the number of ESCRT-III subunits enabled functional specialization of the machinery so that it was able to act at different sites in the complex eukaryotic cell while obeying the same fundamental mechanistic rules that have been conserved over >2 billion years of evolution.

## MATERIALS AND METHODS

### Phylogenetic analysis

Eukaryotic and Asgard ESCRT-III sequences were identified by HMMER (*72*) and BlastP (*73*) searches and retrieved from UniProt (*74*), GenBank (*75*) and InterPro (*76*) databases, with the goal of collecting a broad distribution of ESCRT-III homologs. A total of 266 eukaryotic (33 organisms) and 81 Asgard (39 species) protein sequences were used for this analysis. Sequences were aligned with MAFFT version 7.05 (*77*) with the (L-INS-i) setting. Maximum likelihood trees were inferred using IQTREE2 (2.2.6) (*78*). Model fitting was performed in IQTREE2 for the focal analyses, with the inclusion of additional complex models (LG+C10-C60+F+G) (*79*) to the default list of models. We included 10,000 ultrafast bootstraps replicates (*80*) and optimized weights of mixture models. For the topology tests, constraint trees were built using TreeViewer (*81*) and constrained topologies were inferred using the LG+C20+F+G model in IQTREE2. These constrained topologies were then compared using the approximately unbiased test (*P* = 0.05) (*82*).

### Gene cloning, protein expression, purification, and chemical labeling

Genes coding for Asgard ESCRT-IIIA and ESCRT-IIIB (UniProt accessions A0A1Q9PC75 and A0A1Q9PC98, respectively) were cloned into the pET-28a vector (Novagen) with a His-tag and a plant SUMO domain (bdSUMO from *Brachypodium distachyon*) as N-terminal fusions (*83*, *84*). Plasmids were used to transform *Escherichia coli* Rosetta DE3(pLysS) (Novagen) for recombinant protein expression. Cells were grown in 2xTY at 37°C, and protein expression was induced for 4 hours by the addition at the midlog phase of 0.5 mM isopropyl-β-d-thiogalactoside. Cells were harvested by centrifugation and lysed by sonication, the cleared lysate was loaded onto a Histrap HP 1-ml affinity column (Cytiva) using buffer A [20 mM tris-HCl, pH 8.0, 500 mM NaCl, and 5% (v/v) glycerol], and proteins were eluted by a linear gradient of buffer A containing 500 mM imidazole-HCl (pH 8.0). The N-terminal His-bdSUMO tag was removed by the *B. distachyon* SUMO protease (bdSENP1) (*83*, *84*). After tag removal, the resulting polypeptides are the full-length versions of the Asgard proteins without any extra residues. The untagged samples were further purified using a HiLoad 16/600 Superdex 200 size exclusion column (Cytiva) equilibrated with buffer A. For the fluorescence microscopy experiments, purified proteins were chemically labeled at the N terminus with TFP-Alexa Fluor 488 and/or Alexa Fluor 568 NHS ester (Thermo Fisher Scientific) following the procedure provided by the manufacturer.

### Analytical size exclusion chromatography

A HiLoad 16/600 Superdex 200 size exclusion column (Cytiva) equilibrated with buffer A [20 mM tris-HCl, pH 8.0, 500 mM NaCl, and 5% (v/v) glycerol] was used for analytical size exclusion experiments. The calibration curve is presented in fig. S2B and was established using the following standard proteins (Merck, MWGF1000): carbonic anhydrase (29 kDa), bovine serum albumin (66 kDa), alcohol dehydrogenase (150 kDa), β-amylase (200 kDa), apoferritin (443 kDa), and thyroglobulin (669 kDa).

### LUV and nanorod preparation and generation of filaments on and off membranes

LUVs were prepared by mixing 1,2-dioleoyl-*sn*-glycero-3-phosphocholine (DOPC) and the negatively charged lipid DOPS (Avanti) in chloroform in a molar ratio of 6:4, followed by evaporation and resuspension in buffer A [20 mM tris-HCl, pH 8.0, 500 mM NaCl, and 5% (v/v) glycerol] to a final concentration of 1 to 2 mM. Lipids were then subjected to 10 cycles of vortexing, freezing in liquid nitrogen, and thawing. NTs were prepared following a similar protocol but using a molar ratio of 4 DOPS:2 DOPC:4 d-galactosyl-β1-1′-*N*-nervonoyl-d-erythro-sphingosine (galactosyl ceramide; Avanti) and without the freezing and thawing cycles. Membranes were aliquoted, flash frozen, and kept at −70°C until use.

To generate Asgard ESCRT-III filaments, proteins at 20 μM in buffer A [20 mM tris-HCl, pH 8.0, 500 mM NaCl, and 5% (v/v) glycerol] were dialyzed overnight at 4°C in the absence or presence of 200 to 2000 μM lipids, forming LUVs or NTs. The dialysis buffer was 20 mM bis-tris-HCl (pH 6.0) for Asgard ESCRT-IIIB and 20 mM Hepes-NaOH (pH 7.0) or 20 mM tris-HCl (pH 8.0) in the case of Asgard ESCRT-IIIA with or without membranes, respectively.

### Negative-stain electron microscopy

Negative-stain grids were prepared using a standard protocol (*85*), pipetting 3 to 8 μl of samples on glow-discharged ultrathin carbon film–supported copper grids (EMS CF400-Cu-UL), followed by staining with 2% (w/v) uranyl acetate for 1 min. Data collections were carried out in FEI Tecnai G2 Spirit and Thermo Fisher Scientific Talos L120C LaB6 cathode TEM microscopes operated at 120 keV, equipped with Ultrascan 1000 (Gatan) and CETA (Thermo Fisher Scientific) detectors, respectively.

### Cryo-EM sample preparation and data collection

Asgard ESCRT-IIIB filaments generated in the presence of 6 DOPC:4 DOPS LUVs were applied onto 300-mesh Quantifoil R1.2/1.3 holey carbon gold grids, which were glow discharged for 12 s at 30 mA in an Edwards sputter coater S150B. Aliquots of 2.5 μl were pipetted on the glow-discharged grids, blotted with filter paper for 3 to 7 s and with a force of −15 N, and plunge frozen in liquid ethane using a Vitrobot Mark IV (Thermo Fisher Scientific) at 20°C and 95% humidity. Grids were screened using a Glacios 200-kV microscope equipped with a Falcon III detector (Thermo Fisher Scientific). Cryo-EM movies were acquired at 105,000× magnification (0.826 Å per pixel) on a Titan Krios G3 300-kV microscope (Thermo), equipped with an X-FEG, 100-μm objective aperture, Gatan BioQuantum energy filter (20-eV slit width), and K3 direct electron detector (Gatan) operated in counting mode. A total of 29,600 movies (50 frames, 1 e^−^/Å^2^ per frame) at a defocus range of −0.6 to −2.6 μm were recorded using automated data collection with EPU (Thermo Fisher Scientific). The same dataset was used to solve the structure of Asgard ESCRT-IIIB filaments and membrane-bound arrays.

### Cryo-EM image processing of ESCRT-IIIB filaments

Movies were drift corrected using MotionCorr2 (*86*) implemented in Relion 4.0 (*87*) to generate micrographs. The contrast transfer function (CTF) parameters were estimated using CTFFIND4 (*88*). These micrographs were used to solve the structure of both membrane-unbound filaments and membrane-bound arrays.

Filaments were picked from the micrographs using the filament option in crYOLO 1.7.5 (*89*) with a model that was trained on manually picked micrographs (~100). Manual inspection of the micrographs revealed a 31-Å repeating pattern, which was used to evenly space the picks along the filaments. A total of ~4.4 million particle images were extracted from the coordinates at 2.5 Å per pixel (100-pixel box size) in Relion. 2D classification with 120 classes, “ignore CTF until first peak”, and shifts restricted to 31 Å along the helical axis revealed well-defined classes representing ~760,000 particles. These were used to generate a 3D model de novo using InitialModel in Relion with four classes and imposing helical restrictions as additional arguments (--helix --helix_outer_diameter 130 --ignore_helical_symmetry --helical_keep_tilt_prior_fixed). The map that clearly resembled the 2D class averages was used to perform a symmetry search with relion_helix_toolbox (*90*), which revealed a helical symmetry of 15.5-Å rise and −178.5° twist. A less stringent selection of the 2D classes provided ~4.1 million particles, which were used to refine against the InitialModel map.

The particles and map were imported into CryoSPARC 4.2.1 (*91*) for a Helix Refine job with the “align to symmetry axis” option and dynamic mask, resulting in a 5.2-Å resolution map. Particles were then converted back to Relion using csparc2star.py from pyem (*92*) and extracted to 1.8 Å per pixel (188-pixel box size) with recentering according to the refined angles and shifts. A 3D refinement in Relion using a mask covering 55% of the *z* axis, where helical symmetry parameters were searched and imposed, resulted in a 3.7-Å resolution map. To isolate high-quality particles, a 3D classification without alignment with five classes (*T* = 50) revealed a good class with ~442,000 particles. A 3D refinement of this subset resulted in a 3.5-Å resolution. Per-particle defocus and per-micrograph astigmatism were refined, after which processing was transferred to Relion 5.0 (*93*). Another 3D refinement with Blush regularization (*94*) resulted in a 2.9-Å resolution. Bayesian polishing was subsequently performed using trained parameters (*95*), wherein polished particles were re-extracted at 0.826 Å per pixel (500-pixel box size). 3D refinement with a mask covering 40% of the *z* axis resulted in a 2.9-Å resolution. Another round of defocus and astigmatism refinement and 3D refinement did not notably improve the map. Therefore, a 25% *z*-mask was used for signal subtraction (250-pixel box size), from which a 3D classification without alignment (eight classes, *T* = 4) revealed a slightly better class with ~275,000 particles, which were reverted to the original box size. 3D refinement (40% mask), defocus and astigmatism refinement, and another round of 3D refinement resulted in the final map at a 2.9-Å resolution (15.4-Å rise and −178.5° twist). The map was sharpened with a *B*-factor of −57 Å^2^.

### Model building and refinement of ESCRT-IIIB filaments

The sharpened map was used to build a model de novo using ModelAngelo (*96*). Different segments generated by ModelAngelo were manually stitched together in Coot 0.9.3 (*97*) to generate an asymmetric unit consisting of two monomers. Because of the relatively poor density of α helix α5 and the adjacent region of α1/α2, an AlphaFold2 prediction (*98*) was generated from the interaction using a local installation of ColabFold 1.2.0 (*99*) running MMseqs2 (*100*). The region of high confidence relating to this interface was stitched into each monomer model. The monomer side chains were then manually refined in Coot 0.9.3. For real-space refinement of the polymer, the asymmetric unit was copied to the helically related positions using the sym option in Chimera 1.16 (*101*). Refinement of the polymer was performed in Phenix 1.20 (*102*) by defining two noncrystallographic symmetry groups relating to each of the two monomers in the asymmetric unit. After refinement, symmetry-related copies were removed, and poorly defined side chains were trimmed before model validation. Problematic side chains were manually fixed in Coot before another round of polymer refinement and monomer validation. The map to model Fourier shell correlation was calculated on the polymer. Coloring of the map and model, including residues that interact, was done in ChimeraX 1.7 (*103*).

### Cryo-EM image processing and model building of membrane-bound arrays

Motion-corrected micrographs containing membrane-bound ESCRT-IIIB arrays were picked using a crYOLO (*89*) model trained against membrane edges. An inspection of the micrographs revealed a 30-Å repeating spiked pattern. Therefore, coordinates that are spaced by 30 Å were picked along the membrane edges. An extraction of particle images followed by 2D classification revealed that the membrane signal obscured the alignment of the protofilament arrays. To overcome this, the coordinates were resampled along the membrane edge to 20 Å such that they are out of phase with the 30-Å repeat. Therefore, a rolling 2D average of the particles along any given membrane edge only enhanced the diffuse membrane signal and averaged out the protein signal. This rolling average was subtracted from the micrographs, leaving the arrays intact. Coordinate resampling and micrograph subtraction were performed using scripts originally implemented for microtubule-based processing (*60*). The original coordinates could then be extracted at 2.8 Å per pixel (90-pixel box size) in CryoSPARC 4.2.1 (*91*), and a 2D classification with 50 classes while restricting shifts along the helical axis resulted in classes where the arrays wrapping around the membrane edge were nicely resolved (~579,000 particles). Another round of 2D classification resulted in a subset of ~378,000 particles. These were used for ab initio reconstruction using one class. The resulting map and particles underwent homogeneous refinement (dynamic mask) followed by local refinement of the central five protofilaments, resulting in a map at a 5.8-Å resolution, although map connectivity remained poor at this stage.

Refined particle alignments from CryoSPARC were then converted to Relion using csparc2star.py and extracted to 2.8 Å per pixel (90-pixel box size) with recentering. These underwent 3D refinement in Relion 5.0 (*93*), resulting in a 5.6-Å resolution map. To improve the definition of the protofilaments, the particles were re-extracted away from the very edge of the membrane (1.5 Å per pixel, 192-pixel box size). The central three protofilaments were then refined using Blush regularization (*94*) and helical symmetry (also used for all subsequent refinements). In this case, symmetry relates the adjacent protofilaments, which stack laterally in the *z* axis (30.1-Å rise and 0.1° twist). The resolution at this stage was 6.5 Å, with improved connectivity between α-helical densities. A 3D classification without alignment with four classes (*T* = 4) was then performed, which revealed a good class with ~33,000 particles. These were refined and re-extracted at 1.5 Å per pixel (274-pixel box size), shifting slightly further from the membrane edge again. To remove picks that might relate to the same particle (i.e., those that the software may have inadvertently picked twice on the same membrane edge), coordinates that were within 15 Å of each other were removed. A final 3D refinement of the central four protofilaments resulted in a 6.5-Å resolution (29.9-Å rise and 0.4° twist). The map was sharpened using a *B*-factor of −80 Å^2^.

To build the membrane-bound array model, we used our high-resolution model of the ESCRT-IIIB filament. One protofilament was extracted from the model and flexibly fit into the central protofilament of the membrane-bound array map in Coot 0.9.3 (*97*) using secondary structure restraints. Real-space refinement was performed in Phenix 1.20 before the backbone was trimmed in regions where the map was poorly resolved. To identify the interacting faces, copies of the central protofilament model were generated using the sym command in Chimera 1.16 (*101*). The map to model Fourier shell correlation was calculated on the model that has adjacent protofilament copies. Map and model coloring, including hydrophobicity and electrostatic potential, was performed in ChimeraX 1.7 (*103*).

### Lipid-covered silica bead preparation

SLBs, GUVs, and membrane NTs pulled from GUVs were prepared from lipid lamellae deposited on 40-mm silica beads, as previously described (*104*). DOPC, the negatively charged lipid DOPS, and Atto-647DOPE (1,2-dioleoyl-*sn*-glycero-3-phosphoethanolamine labeled with Atto 647N; Avanti Lipids) were mixed at either 59.95:40:0.05 or 79.95:20:0.05 mol % ratios from lipid stocks purchased from Avanti and dissolved in chloroform to a final concentration of 1 mg/ml. The lipid mixture was then dried in vacuum for no less than 2 hours, allowing complete chloroform evaporation, forming a dried lipid film. The lipid film was hydrated and resuspended in 25 mM Hepes-NaOH buffer at pH 7.4, forming multilamellar vesicles. Subsequently, 10 ml of the suspension was mixed with 1 ml of 40-mm silica beads (Microspheres-Nanospheres), divided into five drops, and placed on a clean parafilm surface. Bead-multilamellar vesicle drops were then dried in vacuum for at least 1 hour until complete evaporation of the buffer.

### SLB membrane preparation and TIRF microscopy on Asgard ESCRT-III protein recruitment

SLBs were prepared as previously described (*104*, *105*). Initially, a coverslip was cleaned in water/ethanol/water for 10 min each using sonication. Following this washing step, coverslips were completely dried using nitrogen gas and plasma cleaned (Harrick Plasma) for 30 s. An Ibidi chamber (sticky-Slide VI 0.4) was then mounted on the coverslip and filled up with working buffer (25 mM Hepes-NaOH and 10 mM MgCl_2_ at pH 6.5). Lipid-covered silica beads (see the “Lipid-covered silica bead preparation” section) were transferred using a 20-ml plastic micropipette tip to the wells used for the experiments. After 10- to 15-min incubation, lipid bilayers were spilled on the coverslip, leading to the formation of the SLBs. Fluorescently labeled proteins (at concentrations and combinations specified in each experiment) were added to the Ibidi’s inlet using a syringe pump withdrawing the bulk volume from the outlet position of the flow chamber. Sequential addition experiment of ESCRT-IIIB and ESCRT-IIIA was performed by first incubating ESCRT-IIIB with the SLBs until the formation of ESCRT-IIIB patches, then washing out bulk ESCRT-IIIB to stop its polymerization before complete membrane surface coverage, and lastly adding ESCRT-IIIA in the incubation chamber. Simultaneous ESCRT-IIIB and ESCRT-IIIA addition was performed by adding both proteins at the same time in the incubation chamber.

TIRF microscopy experiments were performed using an Olympus IX83 wide-field microscope equipped with an Olympus Uapo N 100× 1.49 oil objective and an ImageEM X2 EM-CCD camera (Hamamatsu). The system was controlled by VisiView v4.4.0.11 software (Visitron Systems GmbH).

### Analysis of polymerization rate and maximum surface coverage by ESCRT-IIIB

The fluorescence increase rate for polymerization was obtained from the slope of the linear region of the fluorescence plot profile (Fig. 3B) at the concentrations at which ESCRT-IIIB polymerizes (300, 500, 750, and 1000 nM). Maximum surface coverage by ESCRT-IIIB was obtained by fitting the experimental data from Fig. 3C with a sigmoid function and extracting the value corresponding to the maximum fluorescence intensity at the steady state.

### GUV and NT experiments

GUVs were prepared using lipid-covered silica beads (see the “Lipid-covered silica bead preparation” section), as described earlier (*104*). Lipid-covered silica beads were hydrated in a 1 M trehalose solution for 15 min at 60°C in a home-made humidity chamber and deposited in the microscopy observation chamber. At this stage, growing GUVs were still attached to the silica beads. Therefore, to generate freestanding GUVs, the chamber was gently stirred manually for 1 min, promoting the detachment of GUVs from the silica support beads. Fluorescently labeled protein (concentration and subunit composition specified on each particular experiment) was carefully added after freestanding GUV production. For pulling membrane NTs from freestanding GUVs, closed glass micropipettes were initially prepared using a P-1000 micropipette puller (Sutter Instruments, US). Lipid NTs were produced by direct physical contact between the micropipette and the GUVs. Micropipettes were moved in *xyz* inside the microscopy chamber using a micropositioning system (MP-285, Sutter Instrument, Novato, CA). Fluorescently labeled protein was added immediately before tube pulling to the microscopy chamber (concentration indicated in each experiment). In both cases, fluorescence image acquisition was carried out using an inverted spinning disc microscope assembled by 3i (Intelligent Imaging Innovation), composed of a Nikon base (Eclipse C1, Nikon), a 100× 1.49–numerical aperture oil immersion objective, and an EVOLVE EM-CCD camera (Roper Scientific).

### Measurement of Asgard ESCRT-IIIB relative abundance between curved and flat membranes

The relative abundance of fluorescently labeled ESCRT-IIIB was obtained by extracting the plot profile of the protein signal in the curved and flat regions of the membrane (GUV and pulled lipid NT, respectively). Plot profile data points were then normalized against plot profiles of the fluorescence intensity obtained from the membrane at the exact same regions of interest, as previously used to measure the ESCRT-IIIB fluorescence intensity.

### Quantification of ESCRT-III–induced membrane structural features

Quantification of Asgard ESCRT-IIIA filament thickness after incubation with LUVs and nanorods was performed by measuring the maximum distance between the outer edges of the protein filaments. The relative orientation of the distinct ESCRT-III filaments (either ESCRT-IIIB alone, ESCRT-IIIA alone, or the mixture of both) was obtained by measuring the smaller angle between the membrane longitudinal axis and the filament decorating the membrane (as indicated in Fig. 6A by the arrows). Filament distances from samples with different Asgard ESCRT-III subunit compositions were obtained by measuring the peak distances in their plot profiles perpendicular to the filament longitudinal axis.
