# Supplementary material for: Asgard archaea reveal the conserved principles of ESCRT-III membrane remodeling
Source: Sci Adv. 2025 Feb 7;11(6):eads5255. doi: 10.1126/sciadv.ads5255 (PMC11804906; doi:10.1126/sciadv.ads5255)
Supplement: Supplementary file 1 — Figs. S1 to S7 Table S1 [file sciadv.ads5255_sm.pdf]

Science Advances
AAAS

Supplementary Materials for

Asgard archaea reveal the conserved principles of ESCRT-III membrane remodeling

Diorge P. Souza *et al.*

Corresponding author: Aurélien Roux, aurelien.roux@unige.ch; Buzz Baum, bbaum@mrc-lmb.cam.ac.uk

Sci. Adv. **11**, eads5255 (2025)
DOI: 10.1126/sciadv.ads5255

This PDF file includes:

Figs. S1 to S7
Table S1

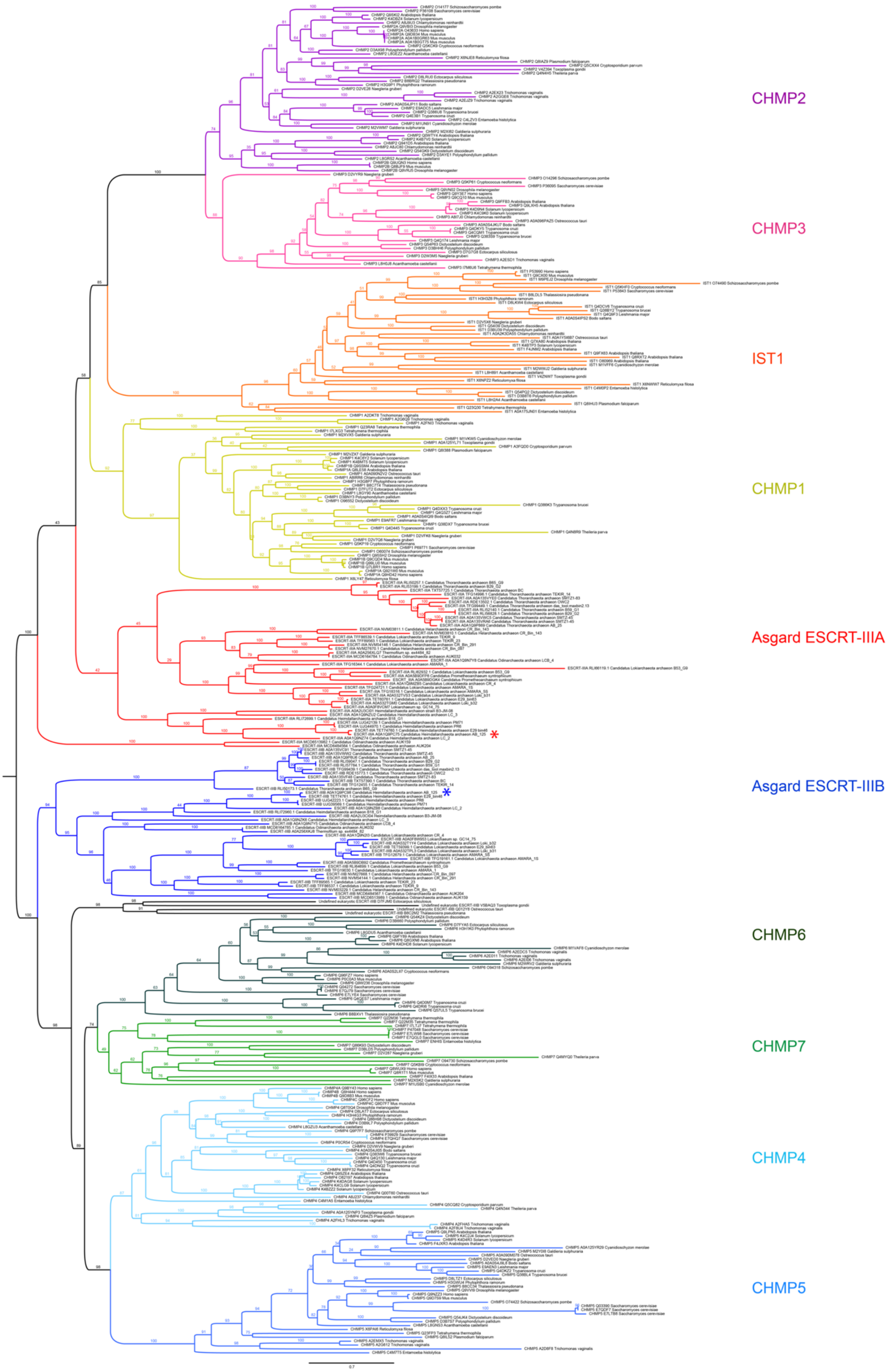
CHMP2
CHMP3
IST1
CHMP1
Asgard ESCRT-IIIA
Asgard ESCRT-IIIB
CHMP6
CHMP7
CHMP4
CHMP5
0.7

Fig. S1. **Complete phylogeny of the eukaryotic and Asgard ESCRT-III subfamilies**. A more detailed tree than shown in Fig. 1A, using an equivalent colour scheme. For each sequence, the corresponding ESCRT-III subfamily, its UniProt or GenBank code, and the species in which it is found are described. Numbers show the relative support from 10,000 bootstrap replicates. Scale bar represents expected substitutions per site. The A- and B-type sequences from Heimdallarchaeota archaeon AB_125, investigated experimentally in this study, are labelled with red and blue asterisks, respectively.

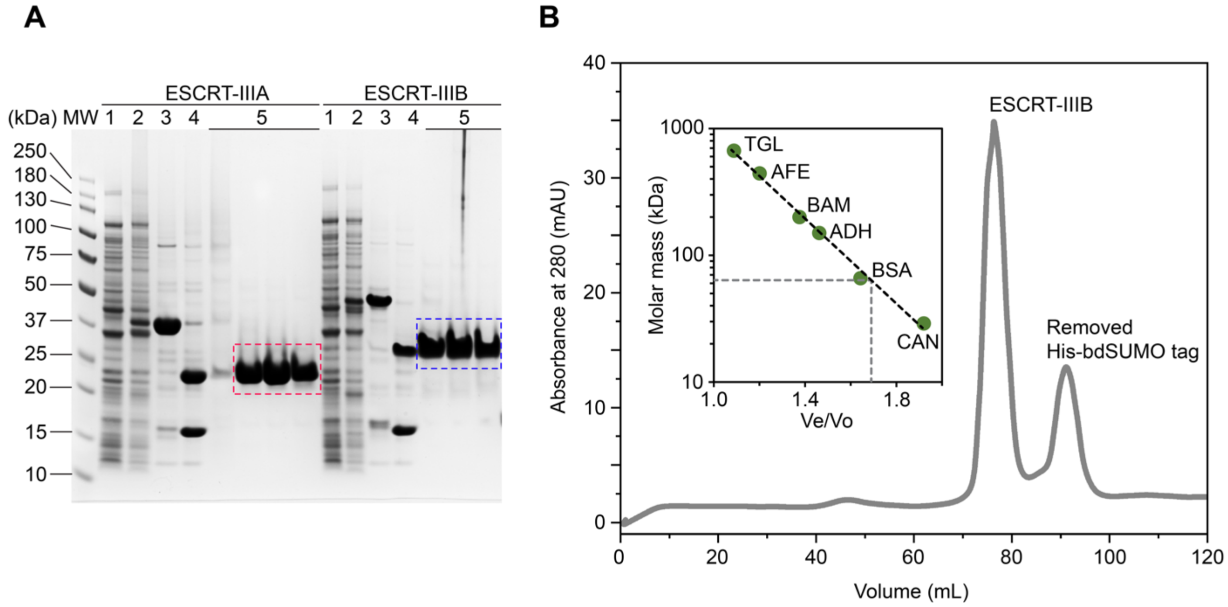

Fig. S2. **Protein purification and analytical size exclusion chromatography**. (**A**) SDS-PAGE of the purification of Asgard ESCRT-IIIA (left) and ESCRT-IIIB (right). MW: molecular weight (in kDa) marker; 1: and 2: *E. coli* cell extract before and after the addition of the inducer, respectively; 3: affinity chromatography fractions; 4: samples after the addition of the bdSUMO protease and tag cleavage; 5: size-exclusion chromatography fractions. Purified ESCRT-IIIA and ESCRT-IIIB are indicated by the red and blue dashed boxes, respectively. (**B**) Superdex 200 16/600 size-exclusion profile of Asgard ESCRT-IIIB (predicted mass of its monomer: 24 kDa). The peak corresponding to the His-bdSUMO tag removed by proteolysis is also indicated. The void volume (V_o) of this column corresponds to approximately 45 mL. Inset: S200 16/600 calibration curve (dashed black line) generated using 6 standard protein markers [green dots: carbonic anhydrase (CAN; 29 kDa), bovine serum albumin (BSA; 66 kDa), alcohol dehydrogenase (ADH; 150 kDa), beta-amylase (BAM; 200 kDa), apoferritin (AFE; 443 kDa) and thyroglobulin (TGL; 669 kDa)]. The dashed grey lines represent the V_e/V_o (V_e: elution volume) and the predicted molar mass (63 kDa) of ESCRT-IIIB. This experiment indicates that this protein does not predominantly form high polymeric forms during purification and is expressed and purified as a dimer or most likely as an elongated monomer.

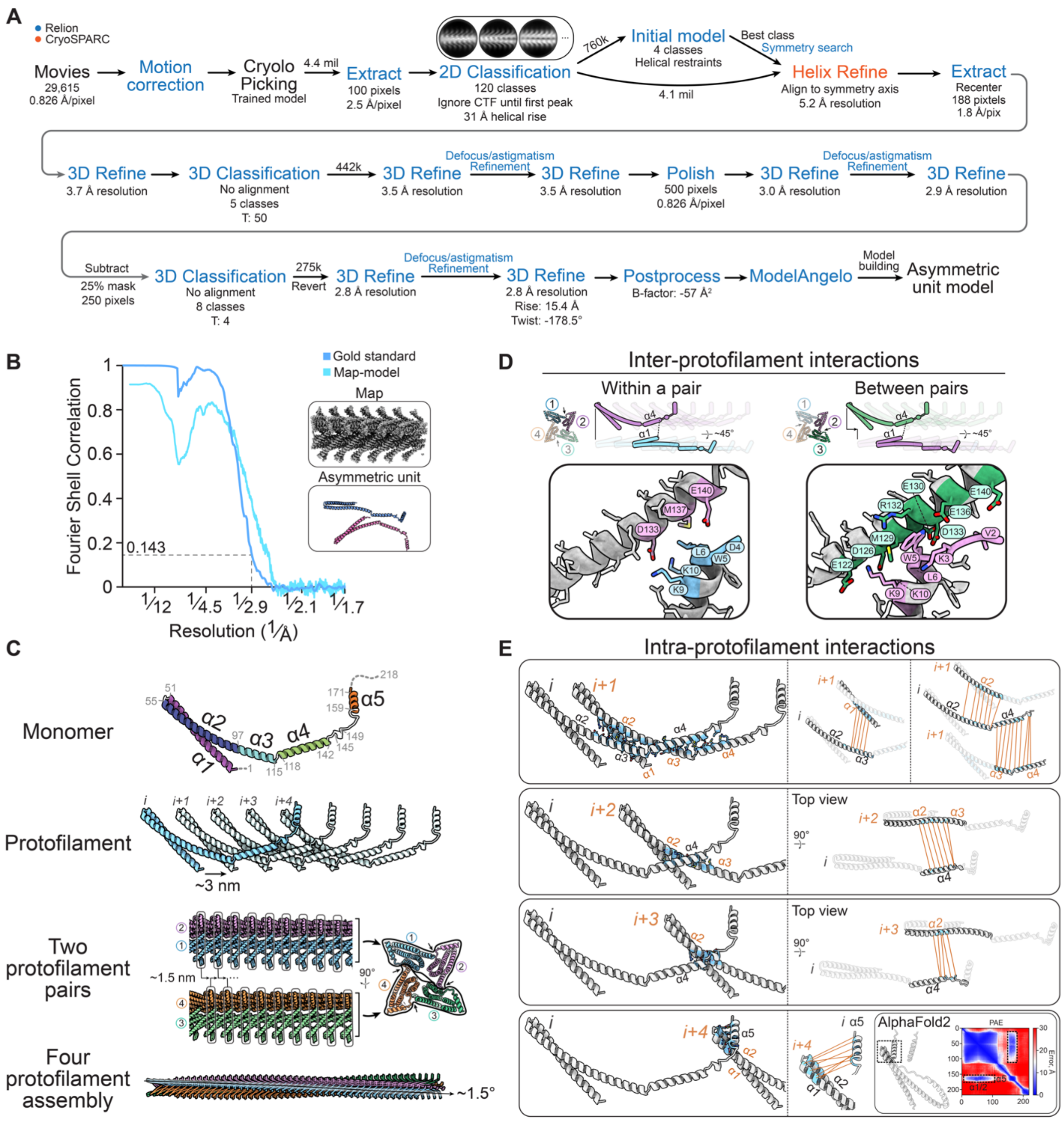

Fig. S3. **Cryo-EM processing and structural characterisation of ESCRT-IIIB filaments**. (**A**) Processing pipeline for single-particle analysis of Asgard ESCRT-IIIB filaments. Relion jobs are labelled in blue and CryoSPARC jobs are labelled in orange. All 3D refinements include helical refinement, where helical parameters are searched and imposed. (**B**) Fourier shell correlation showing the 0.143 cut-off. (**C**) Subunit architecture of Asgard ESCRT-IIIB monomers, protofilaments, and the four-protofilament assembly. Two protofilament pairs are distinguished by the different axial shift of their subunits along the helical axis. A zoomed-out view of the filament shows the ~1.5° supertwist. (**D**) Interaction interface between adjacent protofilaments within a pair (i.e. axially aligned) and between pairs. Note that W5 and D4 sidechains are unresolved and therefore hidden in the left-hand zoom-in. (**E**) Overview of the interaction between adjacent subunits of a protofilament, which includes *i* to *i+1* / *i+2* / *i+3* / *i+4*. Interacting residue backbones are coloured in cyan and interfaces are separated to help visualise the interactions (orange lines). An AlphaFold2 prediction of the *i* to *i+4* interaction is shown along with its predicted aligned error (PAE) plot.

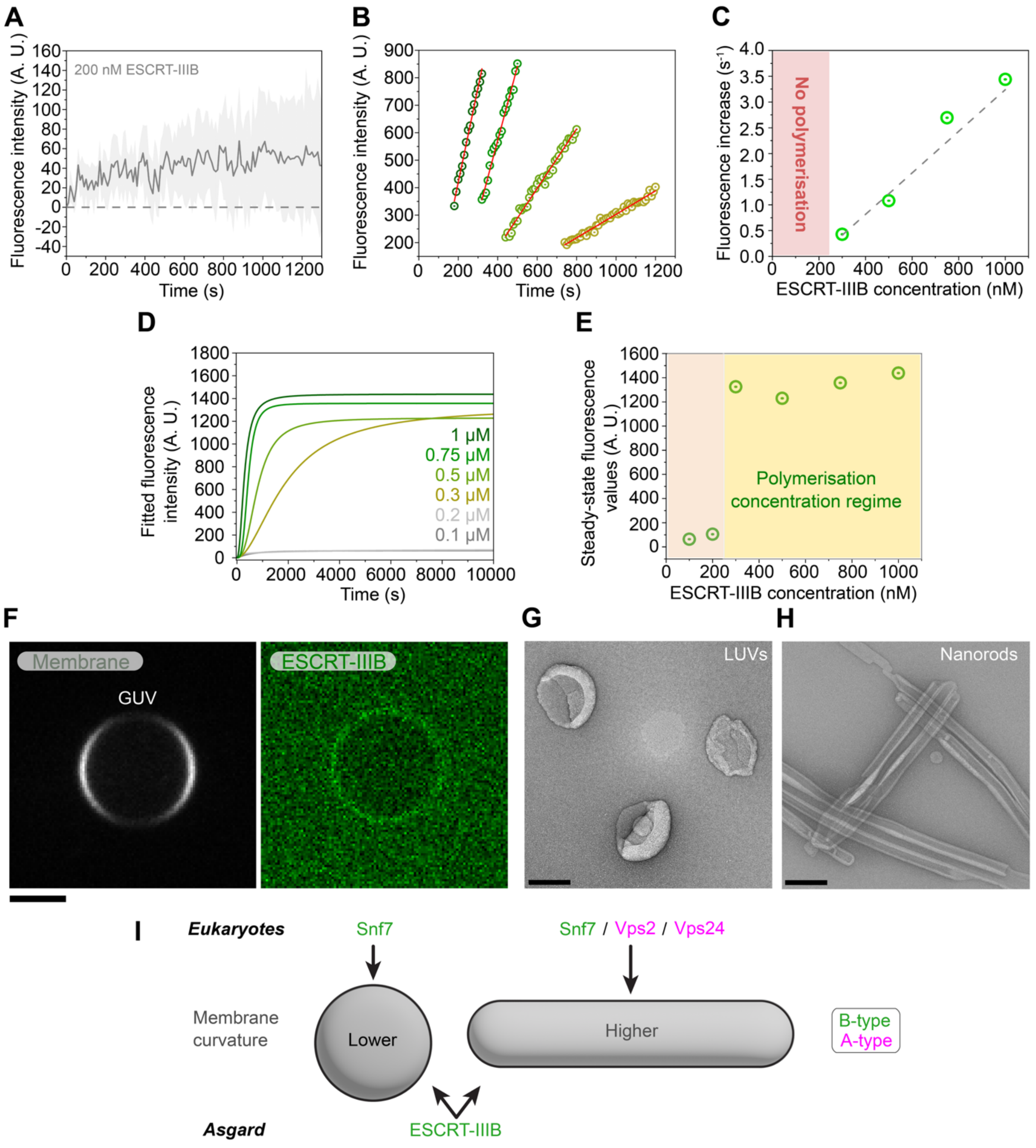

Fig. S4. **Interaction of Asgard ESCRT-IIIB with lipid membranes**. (**A**) Fluorescence intensity time-profile of Asgard ESCRT-IIIB on SLBs below the polymerisation concentration regime (200 nM), lacking the characteristic sigmoidal shape of ESCRT-IIIB polymer assembly which can be seen in Fig. 3B,C (line: mean; shadow: SD; N=9 biological replicates). (**B**) Experimental data points and a line fit for the linear part of the polymerisation regime when ESCRT-IIIB is added to membranes at 300, 500, 750 and 1000 nM (from right to left). (**C**) Graph shows rate of increase in fluorescence intensity versus concentration for experiments in which Asgard ESCRT-IIIB was added to membranes. Data was extracted from the linear part of the polymerisation regime (shown in fig. S4B). The grey dashed line is the line fit, indicating a linear increase of growth rate with increasing Asgard ESCRT-IIIB concentrations in this regime. (**D**) Curves show fitted experimental data shown in Fig. 3C using a sigmoidal function. (**E**) Predicted fluorescence intensity value at steady-state, obtained from fitted curves shown in

fig. S4D. Below a concentration of ~250 nM, Asgard ESCRT-IIIB fails to assemble onto membranes (light pink background). Above this concentration it polymerises on membranes until completely covering the SLB surface (yellow background). (**F**) Fluorescence micrograph of a GUV with slight binding of ESCRT-IIIB insufficient to trigger membrane "crumpling". (**G**)-(**H**), Representative micrographs of (G) nanorods and (H) LUVs visualised by negative stain EM. Scale bars = 120 nm. (**I**) Schematic illustrating the ability of ESCRT-IIIB to adapt to different membrane curvatures, as compared to yeast ESCRT-III proteins where the curvature transition is promoted by the addition of Vps2/Vps24 forming a composite heteropolymer together with Snf7.

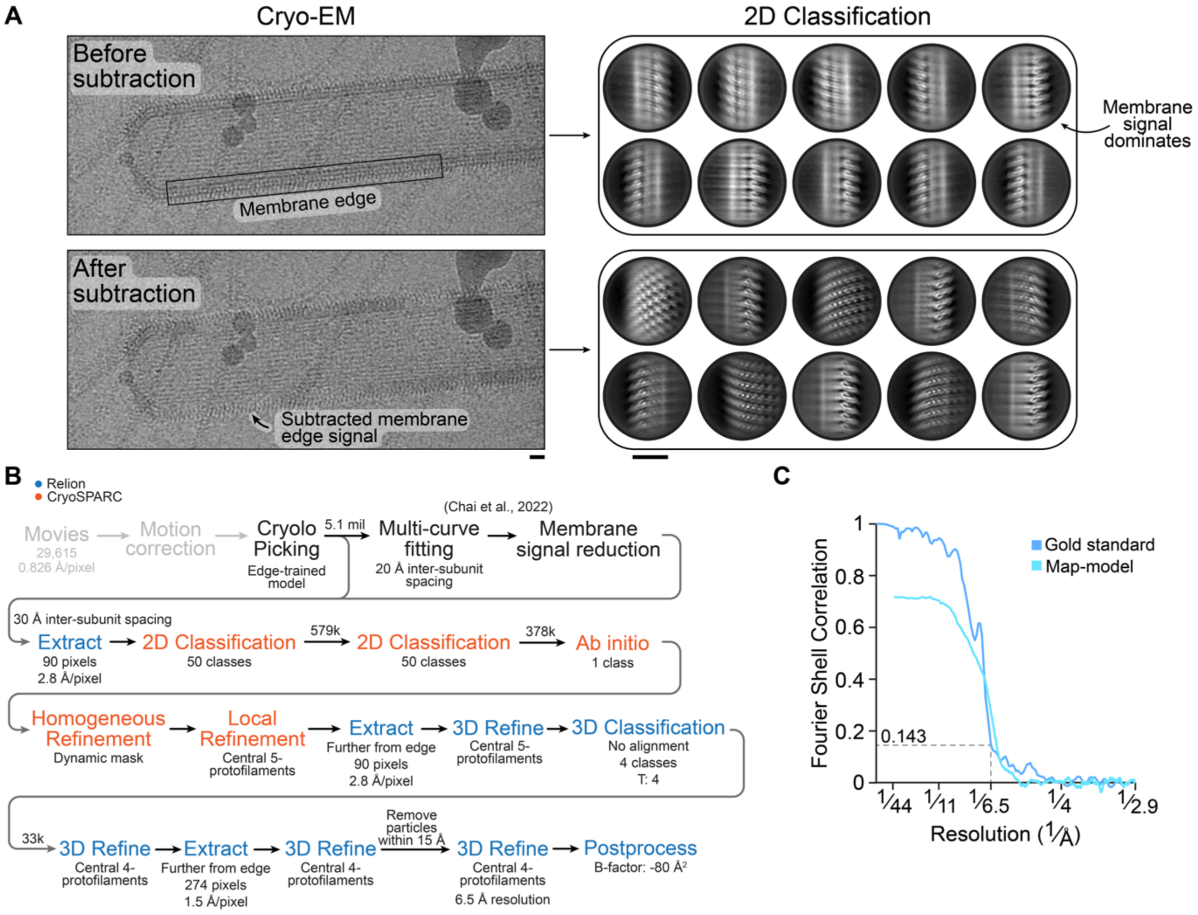

Fig. S5. **Cryo-EM processing of ESCRT-IIIB arrays bound to membranes**. (**A**) An example micrograph before and after subtraction of the membrane signal, as well as representative 2D class averages demonstrating the removal of the dominating signal. Scale bars are 10 nm. (**B**) Processing pipeline for single-particle analysis of membrane-bound arrays. Relion jobs are labelled in blue and CryoSPARC jobs are labelled in orange. (**C**) Fourier shell correlation showing the 0.143 cut-off.

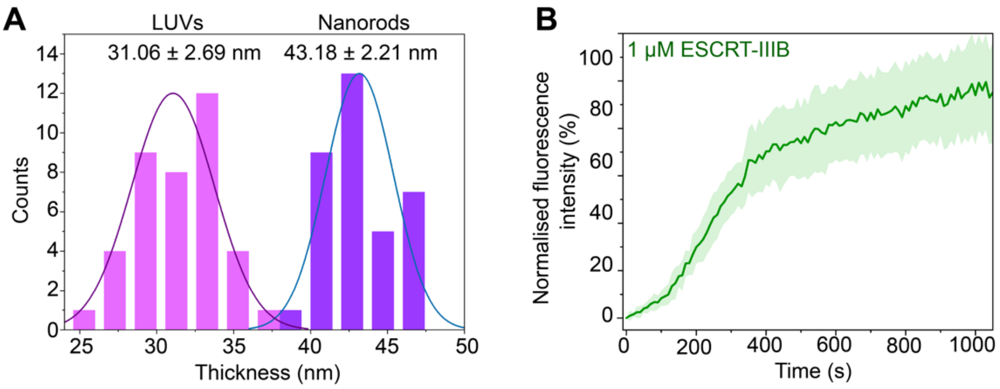

Fig. S6. **Asgard ESCRT-III polymerisation on membrane surfaces**. (**A**) Histogram showing the thickness distribution of ESCRT-IIIA filaments polymerising on LUVs (N=39 biological replicates; 31.06 ± 2.69) and nanorods (N=35 biological replicates; 43.18 ± 2.21) (n = measured filaments; mean ± SD). (**B**) Fluorescence profile of 1 µM Asgard ESCRT-IIIB on SLBs acquired using TIRF microscopy and normalised to its maximum fluorescence intensity. Line: mean; Shadow: SD (N=9 biological replicates).

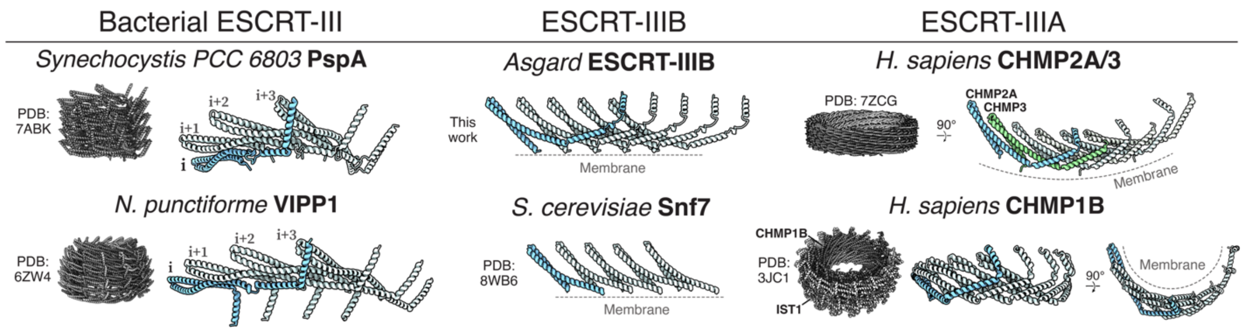

Fig. S7. **Comparison of the ESCRT-III protofilament structures across the tree of life**. The structures of ESCRT-III protofilaments are organised into bacteria, archaea and eukaryotes. Bacterial ESCRT-III includes *Synechocystis PCC 6803* PspA (PDB: 7ABK) (*53*) and *N. punctiforme* Vipp1 (PDB: 6ZW4) (*1*). ESCRT-IIIB includes our structure from Asgard and *S. cerevisiae* Snf7 (PDB: 8WB6) (*47*). ESCRT-IIIA includes *H. sapiens* CHMP2A-CHMP3 (PDB: 7ZCG) (*51*) and *H. sapiens* CHMP1B in complex with IST1 (PDB: 3JC1) (*27*).

Table S1. **Cryo-EM data collection, refinement, and validation statistics of the Asgard ESCRT-IIIB filament and membrane bound array**.

	Filament	Membrane-bound array
**PDB**	9FTL	9FTM
**EMDB**	50748	50749
**Data collection and processing**		
Magnification	105,000	105,000
Voltage (kV)	300	300
Electron exposure ($e^{-}/Å^2$)	50	50
Defocus range (μm)	1.2 – 2.6	1.2 – 2.6
Pixel size (Å)	0.826	0.826
Symmetry imposed	Helical	Helical
Rise (Å)	15.4	29.9
Twist (°)	-178.5	0.4
Initial particle images (no.)	4.4 million	5.1 million
Final particle images (no.)	274,671	27,747
Map resolution (Å)	2.9	6.5
FSC threshold	0.143	0.143
**Refinement**		
Model resolution (Å)	3.1	7.8
FSC threshold	0.5	0.5
Map sharpening *B* factor ($Å^2$)	-57	-80
Model composition		
Non-hydrogen atoms	2,404	2,621
Protein residues	335	527
B factors ($Å^2$)		
Protein	2404/0	2,621/0
R.m.s. deviations		
Bond lengths (Å)	0.005 (0)	0.004 (0)
Bond angles (°)	1.083 (0)	0.946 (0)
Validation		
MolProbity score	0.84	0.8
Clashscore	1.07	1.03
Poor rotamers (%)	0.45	0
Ramachandran plot		
Favored (%)	97.89	98.37
Allowed (%)	2.11	1.63
Disallowed (%)	0	0
